# Heritabilities, proportions of heritabilities explained by GWAS findings, and implications of cross-phenotype effects on PR interval

**DOI:** 10.1007/s00439-015-1595-9

**Published:** 2015-09-18

**Authors:** Claudia Tamar Silva, Jan A. Kors, Najaf Amin, Abbas Dehghan, Jacqueline C. M. Witteman, Rob Willemsen, Ben A. Oostra, Cornelia M. van Duijn, Aaron Isaacs

**Affiliations:** Genetic Epidemiology Unit, Department of Epidemiology, Erasmus University Medical Center, PO Box 2040, 3000 CA Rotterdam, The Netherlands; Doctoral Program in Biomedical Sciences, Universidad del Rosario, Bogotá, Colombia; Department of Genetics (GENIUROS), Escuela de Medicina y Ciencias de la salud, Universidad del Rosario, Bogotá, Colombia; Department of Medical Informatics, Erasmus University Medical Center, Rotterdam, The Netherlands; Department of Epidemiology, Erasmus University Medical Center, Rotterdam, The Netherlands; Department of Clinical Genetics, Erasmus University Medical Center, Rotterdam, The Netherlands; Center for Medical Systems Biology, Leiden, The Netherlands

## Abstract

Electrocardiogram (ECG) measurements are a powerful tool for evaluating cardiac function and are widely used for the diagnosis and prediction of a variety of conditions, including myocardial infarction, cardiac arrhythmias, and sudden cardiac death. Recently, genome-wide association studies (GWASs) identified a large number of genes related to ECG parameter variability, specifically for the QT, QRS, and PR intervals. The aims of this study were to establish the heritability of ECG traits, including indices of left ventricular hypertrophy, and to directly assess the proportion of those heritabilities explained by GWAS variants. These analyses were conducted in a large, Dutch family-based cohort study, the Erasmus Rucphen Family study using variance component methods implemented in the SOLAR (Sequential Oligogenic Linkage Analysis Routines) software package. Heritability estimates ranged from 34 % for QRS and Cornell voltage product to 49 % for 12-lead sum. Trait-specific GWAS findings for each trait explained a fraction of their heritability (17 % for QRS, 4 % for QT, 2 % for PR, 3 % for Sokolow–Lyon index, and 4 % for 12-lead sum). The inclusion of all ECG-associated single nucleotide polymorphisms explained an additional 6 % of the heritability of PR. In conclusion, this study shows that, although GWAS explain a portion of ECG trait variability, a large amount of heritability remains to be explained. In addition, larger GWAS for PR are likely to detect loci already identified, particularly those observed for QRS and 12-lead sum.

**Electronic supplementary material:**

The online version of this article (doi:10.1007/s00439-015-1595-9) contains supplementary material, which is available to authorized users.

## Introduction

Parameters describing electrical activity in the heart, measured by the electrocardiogram (ECG), are important tools for diagnosing, monitoring, and evaluating risk in patients with cardiovascular disease (DeFilippis et al. 2007; Milan et al. 2010; Schwartz and Wolf 1978). ECG measurements, such as PR interval, QRS complex duration, and QT interval, are used for the diagnosis and prediction of cardiac arrhythmias and sudden cardiac death (SCD) (Dekker et al. 2004; Straus et al. 2006; Teodorescu et al. 2011). Myocardial depolarization and repolarization time is measured by the QT interval: the time between the onset of the QRS complex and the end of the T wave. QT shortening or prolongation has been associated with an increased risk for arrhythmias and SCD (Gussak et al. 2000; Straus et al. 2006). PR interval and QRS duration are measures of cardiac conduction time; QRS duration reflects conduction through the ventricular myocardium, while PR interval measures atrial and atrioventricular conduction from the sinoatrial node to the ventricular myocardium, primarily through the atrioventricular node (Smith et al. 2009). ECG calculations of the Sokolow–Lyon index (SL), the Cornell voltage product (CV), and the 12-lead sum QRS product (12LS) have been used as indices of left ventricular hypertrophy (LVH) (Ang and Lang 2008; Molloy et al. 1992), which is a significant predictor of cardiovascular morbidity and mortality (Mayosi et al. 2002; Mutikainen et al. 2009a).

Several studies estimated a high heritability for RR interval (40–98 %) and moderate heritabilities for QT/QTc (25–67 %), PR (34–46 %), and QRS (33–43 %) (Dalageorgou et al. 2008; Eijgelsheim et al. 2009; Haarmark et al. 2011; Havlik et al. 1980; Holm et al. 2010; Im et al. 2009; Kolder et al. 2012; Mutikainen et al. 2009b; Russell et al. 1998; Smith et al. 2009). Only a few studies have estimated heritabilities for ECG indices of left ventricular hypertrophy, 12LS (32 %), SL (36–57 %), and CV (28–32 %) (Mayosi et al. 2002; Molloy et al. 1992; Mutikainen et al. 2009a, b; Shah et al. 2011). Some of these estimates were generated in samples ascertained on the basis of phenotype or from special populations (such as SL and CV) (Havlik et al. 1980).

In recent years, a number of genome-wide association studies (GWASs) for ECG phenotypes identified 65 loci harboring both novel and previously described ECG genes, including two loci influencing electrocardiographic indices related to left ventricular hypertrophy (Arking et al. 2014; Eijgelsheim et al. 2009; Holm et al. 2010; Newton-Cheh et al. 2007, 2009; Nolte et al. 2009; Pfeufer et al. 2009, 2010; Shah et al. 2011; Sotoodehnia et al. 2010). Surprisingly, only a few of the novel loci include genes with established electrophysiological function (such as *ATP1B1* and *PLN* and its negative regulator *PRKCA* (Arking et al. 2014; Barwe et al. 2009; Cerra and Imbrogno 2012; Medeiros et al. 2011)) and only a few have been confirmed through functional analysis (*NDRG4* and *SCN5A*) (Chopra et al. 2010; Qu et al. 2008). These loci typically have small effects, individually accounting for only a small proportion of the variance of these traits. To date, no studies have directly estimated the extent to which these loci explain the trait heritabilities.

The first aim of the present study was to use a large, family-based cohort, not ascertained on the basis of phenotype, to estimate heritabilities for a number of widely used ECG traits. The second was to evaluate the proportion of heritability explained by genetic variants previously identified by GWAS.

## Methods

### Study population

This study was embedded in the Erasmus Rucphen Family study (ERF), a cohort derived from a region in the southwest of the Netherlands. The population was established in the middle of the 18th century by a limited number of founders, has experienced minimal immigration and emigration, and has exponentially increased in size in the last century. The ERF study was instituted in this population to determine the genes underlying quantitative trait variation in humans (Pardo et al. 2005). Interviews at the time of blood sampling were performed by medical practitioners and included questions on education level, smoking status, current medication use, and medical history (Sayed-Tabatabaei et al. 2005). Myocardial infarction was assessed through interview data and ECG measurements. Height and weight were measured with the participant in light underclothing and body mass index (kg/m^2^) was computed. Blood pressure was measured twice on the right arm in a sitting position after at least 5 min rest, using an automated device (OMRON 711, Omron Healthcare, Bannockburn, IL, USA). The average of the two measures was used in the analyses. Hypertension was defined through the use of antihypertensive medication and/or through the assessment of blood pressure measurements according to the World Health Organization guidelines (individuals with BP ≥140/90 mmHg should be regarded as hypertensive) (1999; Mourad 2008; Tin et al. 2002). The Medical Ethics Committee of the Erasmus University Medical Center approved the ERF study protocol and all participants, or their legal representatives, provided written informed consent.

### ECG interpretation and measurement

Examinations included 12-lead ECG measurements. A 10-s 12-lead ECG (on average, 8–10 beats) was recorded with an ACTA-ECG electrocardiograph (Esaote, Florence, Italy) with a sampling frequency of 500 Hz. Digital measurements of the ECG parameters were made using the Modular ECG Analysis System (MEANS) (van Bemmel et al. 1990). In brief, MEANS operates on multiple simultaneously recorded leads, which are transformed to a detection function that brings out the QRS complex and the other parts of the signal. MEANS determines common onsets and offsets for all 12 leads together on one representative averaged beat, with the use of template matching techniques. The measurement and diagnostic performance of MEANS have been extensively evaluated, both by the developers and by others (de Bruyne et al. 1997; Willems et al. 1987, 1991) The MEANS criteria for MI are mainly based on pathological Q waves, QR ratio, and R wave progression (van Bemmel et al. 1990). A cardiologist, specialized in ECG methodology, ascertained the final diagnosis of MI.

MEANS was used to measure several ECG parameters (QRS, PR, and QT) and the three LVH proxies (SL, CV, and 12LS). Sokolow–Lyon was defined as the sum of the S wave in V1 plus the R wave in V5 or V6, Cornell as the sum of R in aVL and the S in V3, and 12-lead as the sum of R–S in all 12 leads; these three voltages were then multiplied by QRS duration to obtain voltage-duration products as an approximation of the area under the QRS complex (Casale et al. 1985; Siegel and Roberts 1982; Sokolow and Lyon 1949). QT interval was adjusted for heart rate using Bazett’s formula (Bazett 1920; Roguin 2011). All traits were adjusted for sex, age, BMI, height and heart rate (with the exception of QT), and rank transformed prior to analysis.

### Genotyping and SNP selection

Genotyping in ERF was performed using Illumina 318/370 K, Affymetrix 250 K, and Illumina 6 K micro-arrays. Individuals were excluded for excess autosomal heterozygosity, mismatches between called and phenotypic gender, and if there were outliers identified by an IBS clustering analysis. The exclusion criteria for SNPs were Hardy–Weinberg equilibrium (HWE) *P* ≤ 10^−6^ or SNP call rate ≤98 %. After this quality control, measured genotypes which had minor allele frequencies >1 % were used to impute ~2.5 million autosomal SNPs with the CEU samples from HapMap release 22 (build 36) as a reference panel with MACH version 1.0.16 (Li et al. 2010). GWAS for ECG traits have identified 71 index SNPs in 65 loci associated at the conventionally accepted significance threshold (*P* < 5.0 × 10^−8^) (Arking et al. 2014; Eijgelsheim et al. 2009; Holm et al. 2010; Marroni et al. 2009; Newton-Cheh et al. 2005, 2007, 2009; Nolte et al. 2009; Pfeufer et al. 2009, 2010; Shah et al. 2011; Sotoodehnia et al. 2010) (catalog of published GWAS: http://www.genome.gov/gwastudies/). These SNPs were extracted from our imputed dataset for further analysis, with the exception of a single QRS SNP (rs991014). If multiple SNPs in a given locus were described in the GWAS, only the SNP with the lowest *P* value was selected for inclusion.

### Statistical analysis

Individuals were excluded from analysis if their ECG showed evidence of atrial fibrillation, myocardial infarction, left or right bundle branch block, or atrioventricular block. Additional exclusion criteria consisted of pacemaker implantation, Wolff–Parkinson–White syndrome, pregnancy, and use of Type I or III antiarrhythmic medications or digoxin, which may shorten the QT interval (Eijgelsheim et al. 2009). Individuals with QRS >120 ms were excluded from the QRS, QT, and LVH proxy analyses. Those with PR ≥320 or ≤80 ms were excluded from the PR analyses. Those with QRS axis >90 or <−30 were excluded from the LVH proxy analyses. These exclusions were implemented to keep our data consistent with previous and ongoing GWAS.

Heritability estimates were obtained using a variance component approach based on maximum likelihood procedures implemented in the SOLAR (Sequential Oligogenic Linkage Analysis Routines) software package (http://www.sfbr.org/solar/index.html). A narrow-sense heritability estimate (*h*^2^) represents the fraction of variation in a trait attributable to additive genetic factors. To determine the proportion of variance due to genotypes associated with ECG trait variability, narrow-sense heritabilities were computed with and without genotypic data (Isaacs et al. 2007); comparison of the log likelihoods of these models using likelihood ratio tests allowed us to assess the significance of the differences. Heritabilities were calculated for each trait (QRS, QT, PR, SL, CV, 12LS) using three adjusted models. The first model adjusted only for non-genetic covariates, the second model included GWAS SNPs specific for each trait, and the third model included all of the ECG-associated SNPs. For the LVH proxies, only two SNPs for 12LS have been reported with genome-wide significance; since these measures are QRS products, the QRS SNPs were included in model 2 for these traits.

Inbreeding coefficients, which represent the level of consanguinity between a subject’s parents, were calculated as previously described (Isaacs et al. 2007). To analyze the impact of inbreeding on the ECG traits, inbreeding coefficient quartiles were included in the SOLAR models. People with zero inbreeding were classed as “0”; the people with non-zero inbreeding were divided into quartiles. The quartiles were used because of the large skew in the distribution.

## Results

After exclusions, 1396–1474 phenotyped and genotyped ERF participants were available for analysis. Table 1 shows descriptive statistics for a number of traits in the study population. The average age of the cohort was 47.5 (±13.8) years and 40 % were men. The population tends toward being overweight, with a mean BMI of 26.7 (±4.5) kg/m^2^. A large number, nearly 30 %, were hypertensive. The median (inter-quartile range) of the pair-wise kinship coefficients for the analyzed sample was 0.004 (0.007); the number of pairs for a broad range of kinship levels are presented in Supplementary Table 1. The median (inter-quartile range) of the inbreeding coefficient was 0.003 (0.009). Correlations between the analyzed traits are presented in Table 2. The correlations between PR, QRS, and QT were modest, especially after adjustment for covariates. Correlations between the LVH proxies, particularly 12LS and SL, were stronger; these measures were also moderately correlated with QRS, a component of each.

**Table 1:** Descriptive statistics of the study population (*N* = 1474)

	Mean (SD)	Minimum	Maximum
Males	597 (40 %)	**–**	**–**
Age (year)	47.2 (13.9)	16.6	81.4
BMI (kg/m^2^)	26.6 (4.5)	15.5	48.6
Height (cm)	166.6 (9.0)	143.6	196.5
Weight (kg)	74.0 (14.8)	41.9	154.7
SBP (mmHg)	136.0 (19.5)	85.5	217.0
DBP (mmHg)	79.8 (9.8)	54.5	120.0
Hypertension	629 (42 %)	–	–
QRS (ms)	97.0 (10.0)	68	120
QT (ms)	397.4 (27.7)	300	520
Heart rate (bpm)	63.0 (10.5)	35	120
PR (ms)	152.3 (22.1)	92	308
SL (mm ms)	2316 (680.2)	1040.0	5288.5
CV (mm ms)	1172.6 (498.3)	118.7	3953.0
12LS (mm ms)	13,670 (3551.6)	5485	32,550

Values presented are mean (SD) or *N* (%)

*BMI* body mass index, *SBP* systolic blood pressure, *DBP* diastolic blood pressure, *SL* Sokolow–Lyon index, *CV* Cornell product, *12LS* 12-lead sum product

**Table 2:** Pearson’s correlations between ECG traits

	QRS	QT	PR	SL	CV	12LS
QRS	1	0.225	0.144	0.376	0.468	0.532
QT	0.152	1	0.229	0.108	0.081	0.089
PR	0.007	0.105	1	0.082	0.169	0.117
SL	0.253	0.014	0.001	1	0.289	0.803
CV	0.382	0.053	0.04	0.178	1	0.564
12LS	0.409	0.034	0.019	0.741	0.484	1

Above the diagonal: unadjusted correlations. Correlations are significant at the 0.01 level (2-tailed). Below the diagonal: adjusted correlations (adjusted for age, sex, body mass index, height and heart rate)

The heritability for heart rate-adjusted QT, prior to the inclusion of SNP information, was 36 % (*P* = 1.14 × 10^−8^). There was no evidence for recessive effects, as the inclusion of inbreeding coefficient did not alter the heritability estimates. The inclusion of SNPs specifically identified for QT (model 2) explained approximately 4 % of the trait’s heritability (leaving 96 % unexplained) (Table 3). A significant difference between model 1 and 2 was observed (*P* = 2.58 × 10^−4^). The additional inclusion of SNPs identified in GWAS of other ECG phenotypes further decreased the heritability by 15 %, although this difference (between model 2 and 3) was not significant (*P* = 0.15).

**Table 3:** Heritability (*h* ^2^) of ECG measurements

	Model 1		Model 2					Model 3			
	*h* ^2^ (SD)	*P*	*h* ^2^ (SD)	*P*	Δ*h* ^2^*	$P_{{\varDelta h^{2} }}$	n SNPs ^REFS^	*h* ^2^ (SD)	*P*	Δ*h* ^2^**	$P_{{\varDelta h^{2} }}$
QRS	0.34 (0.06)	2.32 × 10^−9^	0.28 (0.06)	1.30 × 10^−6^	0.06	2.6 × 10^−3^	21^a, b^	0.27 (0.07)	1.06 × 10^−5^	0.01	0.28
QT	0.36 (0.07)	1.14 × 10^−8^	0.34 (0.07)	1.00 × 10^−7^	0.02	2.5 × 10^−4^	36^a, c–f^	0.29 (0.07)	1.17 × 10^−5^	0.05	0.15
PR	0.40 (0.06)	4.13 × 10^−11^	0.39 (0.06)	1.31 × 10^−10^	0.01	2.6 × 10^−4^	9^a, g, h^	0.37 (0.07)	5.06 × 10^−9^	0.02	1.0 × 10^−3^
12LS	0.49 (0.06)	4.60 × 10^−16^	0.46 (0.06)	1.44 × 10^−14^	0.03	5.7 × 10^−3^	23^b, i^	0.44 (0.07)	3.71 × 10^−12^	0.02	0.15
CV	0.34 (0.07)	7.44 × 10^−9^	0.35 (0.07)	5.20 × 10^−9^	−0.002	6.3 × 10^−5^	21^b^	0.35 (0.07)	1.13 × 10^−8^	−0.005	0.17
SL	0.46 (0.07)	1.00 × 10^−13^	0.44 (0.07)	2.74 × 10^−12^	0.02	0.42	21^b^	0.43 (0.07)	6.76 × 10^−11^	0.01	0.14

Model 1: adjusted for age, sex, body mass index, height and heart rate. Model 2: adjusted for age, sex, body mass index, height, heart rate and SNPs associated with each trait. Model 3: adjusted for age, sex, body mass index, height, heart rate and SNPs associated with all traits (65 in total)

*h* ^2^ heritability, *n SNPs* ^*REFS*^ number of SNPs and references, *SL* Sokolow–Lyon index, *CV* Cornell product, *12LS* 12-lead sum product

^a^Holm et al. (2010)

^b^Sotoodehnia et al. (2010)

^c^Arking et al. (2014)

^d^Marroni et al. (2009)

^e^Newton-Cheh et al. (2009)

^f^Pfeufer et al. (2009)

^g^Newton-Cheh et al. (2007)

^h^Pfeufer et al. (2010)

^i^Shah et al. (2011)

**Fig. 1:**
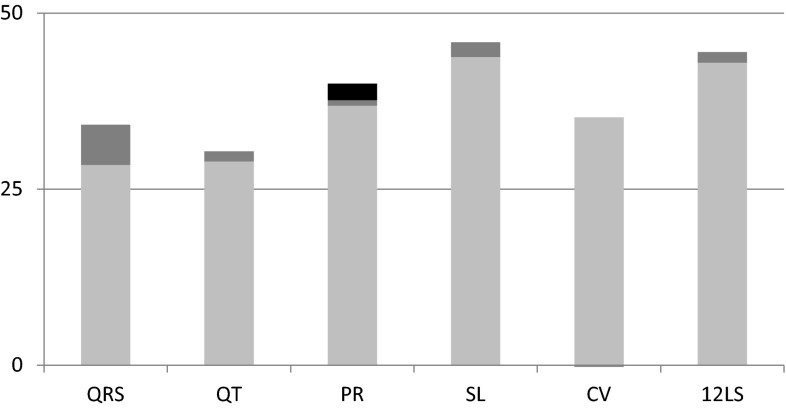
Heritability (*h* ^2^) of ECG measurements. The *height of the bar* indicates trait heritability. The proportion of unexplained heritability is in *light gray* and the proportion of heritability explained by trait-specific SNPs is depicted in *dark gray*. The additional proportion of the explained heritability of PR due to the inclusion of all ECG GWAS SNPs is in *black*

The heritability of QRS was similar to that of QT, 34 % (*P* = 2.32 × 10^−9^). QRS-specific GWAS SNPs explained 17 % of the heritability of QRS (*P* = 1.30 × 10^−6^). Inclusion of non-QRS ECG SNPs did not further explain the heritability (*P* = 0.28). Two percent of the heritability of PR (40 %, *P* = 4.13 × 10^−11^) could be explained by the inclusion of known PR variants (*P* = 2.64 × 10^−4^). The inclusion of SNPs associated with the other ECG phenotypes explained a further 6 % of PR heritability, reducing it to 37 % (*P* = 1.00 × 10^−3^) (Table 3; Fig. 1). To investigate which set of SNPs might offer additional explanatory power for PR, comparisons were made with the addition of the QRS SNPs, the QT SNPs, and the 12LS SNPs in turn. This analysis determined that the majority of the additional PR heritability explained was due to the QRS SNPs (5 %, *P* = 7.09 × 10^−4^), while the two 12LS SNPs explained an additional 0.5 % (*P* = 4.03 × 10^−4^).

Heritability estimates were also calculated for three LVH proxies (SL, CV, and 12LS); since only two GWAS associations (at *P* < 5 × 10^−8^) are known for these traits, and since QRS is a crucial component of all three, QRS SNPs were included in model 2 for each of these measures. 12LS showed the highest heritability of these outcomes (49 %, *P* = 4.6 × 10^−16^), while CV showed the lowest (34 %, *P* = 7.44 × 10^−9^). The heritability estimate for SL was 46 % (*P* = 1.00 × 10^−13^); inclusion of QRS SNPs did not significantly alter this estimate (*P* = 0.42), nor did the inclusion of all ECG SNPs (*P* = 0.15). The inclusion of the two 12LS SNPs and the QRS SNPs explained 4 % of the heritability of 12LS (*P* = 5.77 × 10^−3^). The inclusion of the remainder of the SNPs did not further explain the heritability (*P* = 0.15). For CV, the inclusion of the QRS SNPs increased the estimate slightly, but significantly (0.7 %, *P* = 6.34 × 10^−5^), while the inclusion of all SNPs did not influence the heritability (*P* = 0.18). The additional inclusion of inbreeding coefficient did not impact any of these models (data not shown).

## Discussion

In the present work, we estimated the heritability of ECG traits (QT, QRS, and PR) and ECG-derived LVH indices (SL, CV, and 12 LS). Highly significant estimates of heritability, ranging from 0.34 to 0.49, were observed for all traits, several of which needed replication in a population-based study. The inclusion of known trait-specific GWAS loci explained a fraction of the heritability of each trait except for SL and CV (17 % for QRS, 4 % for QT, 2 % for PR, 4 % for 12LS). Inclusion of all ECG-associated SNPs further explained an additional proportion of the heritability for PR (6 %), clearly suggesting cross-phenotype effects for some loci.

This study benefits from a large, well-characterized, family-based population, selected on the basis of genealogy and not phenotype. It is well powered for this type of analysis and is not biased due to phenotypic selection for, as an example, cardiovascular disease. The complex genealogy allowed for the assessment of inbreeding and, furthermore, accounts for relatedness within families.

Despite these strengths, there are also limitations to this study. One is that ERF was part of the discovery analyses for the QRS and QT GWAS. However, ERF accounts for only a small proportion of those efforts, making it unlikely that over-fitting has a large impact on these findings. Discovery of genetic variants is still in progress. In all likelihood, larger GWAS efforts will lead to the identification of additional SNPs and, therefore, larger proportions of explained heritability. Finally, medication use, abundant in the population, may directly affect variability in ECG measurements. Some medications are known to induce such effects (Ahnve and Vallin 1982; Malik 2004), although, with the exception of the antiarrhythmics (which were excluded in these analyses), these effects are typically small or not well characterized. Moreover, any medication effects should be randomized across genotype groups, and, therefore, unlikely to affect these results.

Heritability estimates for QT between 60 and 67 % have been reported in twin studies (Dalageorgou et al. 2008; Haarmark et al. 2011). Our estimate is substantially lower, but similar to that of another population-based family cohort, the Framingham study (35 %) (Newton-Cheh et al. 2005). This may be due to the inclusion of distant relatives in our study that share fewer household-based environmental factors (Sleegers et al. 2007). Our heritability estimate for QRS is higher than in the previous reports that did not find statistical significance (Havlik et al. 1980; Russell et al. 1998; Smith et al. 2009). Modest sample sizes or poor precision in the QRS measurements may have hindered those earlier studies (Havlik et al. 1980; Russell et al. 1998; Smith et al. 2009). Our estimate is somewhat lower than those reported in older women and a Chinese population (Eijgelsheim et al. 2009; Mutikainen et al. 2009b), but similar to that reported in an Icelandic population (Holm et al. 2010). Our findings for PR heritability are similar to those previously reported (Eijgelsheim et al. 2009; Smith et al. 2009). Among the LVH indices, our SL heritability estimate is less than previously reported in older women (Mutikainen et al. 2009b) and corresponds well to the estimate provided by Mayosi et al. (2002) (~40 %) and Shah et al. (2011) (~39 %). With respect to CV, our estimate corresponds with previous estimates ranging from 23 to 40 % (Mayosi et al. 2002; Shah et al. 2011). For 12LS, our estimate was higher (0.46 %) than previously reported (0.32 %) (Shah et al. 2011).

This is the first study that provides direct estimates of the proportion of heritability attributable to common variants discovered by GWAS. The heritability explained is particularly low for PR and SL, a finding that is not uncommon for complex traits (Manolio et al. 2009). For QRS (17 %), a substantial portion of trait heritability is explained by trait-specific GWAS variants, while for the other traits the proportion is more modest. At the same time, our study shows that large percentages remain unexplained (83 % for QRS, 96 % for QT, 94 % for PR, 94 % for 12LS, 96 % for SL, 100 % CV). These percentages correspond to the “missing heritability”. Several plausible reasons might explain this “missing heritability”, including the overestimation of the heritability of these complex traits and the underestimation of the effects of common alleles identified through GWAS. It should also be noted that we have only studied the percentage of variance explained by one common variant in each locus. When more variants, including rare variants, are taken into account, these loci may explain a larger proportion of the heritability, as some loci are likely to include more than one independent association. Epigenetic modifications, regulated in part by microRNAs through regulation of DNA methyl transferases and histone deacetylases, are often dynamic and influenced by the environmental factors and may play a role. Finally, gene × gene interactions (epistasis) and gene × environment interactions might explain another portion of the heritability of these traits (Manolio et al. 2009; Marian 2012).

The finding that the addition of SNPs identified for another trait to the trait-specific SNPs explained an additional portion of heritability (particularly for the QRS SNPs and PR) strongly suggests the presence of variants with effects across these traits. These cross-phenotype effects are a common phenomenon in complex trait genetics (Solovieff et al. 2013). Known associations reinforce this notion; *TBX5*, for example, has been associated with QRS, PR, and QT (Holm et al. 2010), while *SCN5A* has been associated with both QRS and PR intervals (Pfeufer et al. 2010). This type of cross-phenotype effect was described by Sotoodehnia et al. (2010) who found several QRS loci previously associated with PR or QT intervals, including *PLN, TBX5/3,* and *SCN10A*/*5A*. It is of interest that cross-phenotype effects in particular decreased the heritability of PR.

In conclusion, we report heritability estimates for a number of ECG traits, including three LVH proxies. The incorporation of genotype information allowed for direct estimates of the impact of known GWAS SNPs on ECG trait heritabilities, and indicated that a high proportion of the genetic variability remains to be explained: the so-called “missing heritability”. The inclusion of SNPs identified in GWAS of other ECG phenotypes further increased the amount of PR heritability that could be explained, clearly suggesting that GWAS variants identified for other ECG phenotypes (QRS and 12LS, in particular) influence PR, despite failing to achieve genome-wide significance in PR GWAS to date. Increasing GWAS sample sizes, searching for cross-phenotype effects, and identifying less common variants are likely to increase the explicable portion of ECG trait heritability.

## Electronic supplementary material

Below is the link to the electronic supplementary material.

Supplementary material 1 (DOCX 19 kb)
