# Supplementary material for: Heritabilities, proportions of heritabilities explained by GWAS findings, and implications of cross-phenotype effects on PR interval
Source: Hum Genet. 2015 Sep 18;134:1211–9. doi: 10.1007/s00439-015-1595-9 (PMC4628620; doi:10.1007/s00439-015-1595-9)
Supplement: Supplementary file 1 — Supplementary material 1 (DOCX 19 kb) [file 439_2015_1595_MOESM1_ESM.docx]

**Heritabilities, proportions of heritabilities explained by GWAS findings, and implications of cross-phenotype effects on PR interval**

Short Title:  **Proportion of ECG heritabilities explained by GWAS**

Claudia Tamar Silva, M.Sc.^1,2,3^, Jan A. Kors, Ph.D.^4^, Najaf Amin, Ph.D.^1^, Abbas Dehghan, M.D., Ph.D.^5^, Jacqueline C.M. Witteman, Ph.D.^5^, Rob Willemsen, Ph.D.^6^, Ben A. Oostra, Ph.D. ^1,7^, Cornelia M. Van Duijn, Ph.D.^1,7^, Aaron Isaacs, Ph.D.^1,7^

**Affiliations**

^1^Genetic Epidemiology Unit, Department of Epidemiology, Erasmus University Medical Center, Rotterdam, the Netherlands

^2^Doctoral Program in Biomedical Sciences, Universidad del Rosario, Bogotá, Colombia

^3^Department of Genetics (GENIUROS), Escuela de Medicina y Ciencias de la salud. Universidad del Rosario, Bogotá, Colombia.

^4^Department of Medical Informatics, Erasmus University Medical Center, Rotterdam, the Netherlands

^5^Department of Epidemiology, Erasmus University Medical Center, Rotterdam, the Netherlands

^6^Department of Clinical Genetics, Erasmus University Medical Center, Rotterdam, the Netherlands

^7^Center for Medical Systems Biology, Leiden, the Netherlands

**Corresponding Author**

Aaron Isaacs, PhD

Genetic Epidemiology Unit, Dept. of Epidemiology

Erasmus Medical Center

PO Box 2040

3000 CA Rotterdam

the Netherlands

E-mail: [aaron.isaacs@gmail.com](mailto:aaron.isaacs@gmail.com)

Telephone: +31 10 703 81 25

Fax: +31 10 704 46 57

**Supplementary Table 1. Distribution of kinships in the analyzed ERF sample**

| Kinship Coefficient | φ ≥ 1/4 | 1/4 > φ ≥ 1/8 | 1/8 > φ ≥ 1/16 | 1/16 > φ ≥ 1/32 | 1/32 > φ ≥ 1/64 | 1/64 > φ ≥ 1/128 | 1/128 > φ ≥ 1/256 | 1/256 > φ ≥ 1/512 | 1/512 > φ ≥ 1/1024 | 1/1024 > φ ≥ 1/2048 | 1/2048 > φ ≥ 1/4096 | φ < 1/4096 |
| --- | --- | --- | --- | --- | --- | --- | --- | --- | --- | --- | --- | --- |
| Number of Pairs | 1366 | 1393 | 3998 | 10363 | 61615 | 218893 | 270742 | 170425 | 75382 | 59821 | 55380 | 146950 |

φ: Kinship coefficient.
